# A Proposed Algorithm for the Management of Patients with Cardiogenic Shock Based on Contemporary Knowledge and Gaps in Evidence

**DOI:** 10.3390/jcdd12120489

**Published:** 2025-12-11

**Authors:** Aidonis Rammos, Christos D. Floros, Ioannis Tzourtzos, Ilektra E. Stamou, Petros Kalogeras, Ioanna Samara, Konstantinos C. Siaravas, Vasileios Bouratzis, Aris Bechlioulis, Xenofon M. Sakellariou, Katerina K. Naka, Lampros K. Michalis

**Affiliations:** 1 Second Department of Cardiology, University Hospital of Ioannina, 45500 Ioannina, Greeceioannistzourtz@gmail.com (I.T.); pkalog90@yahoo.com (P.K.); v.bouratzis@gmail.com (V.B.); md02798@yahoo.gr (A.B.); xensakel@gmail.com (X.M.S.);; 2 Service de Cardiologie, Hopitaux du Pays du Mont Blanc, 74700 Sallanches, France; 3 First Department of Cardiology, University Hospital of Ioannina, 45500 Ioannina, Greece

**Keywords:** cardiogenic shock, acute myocardial infarction, heart failure, mechanical circulatory support, mortality

## Abstract

Cardiogenic shock (CS) is a heterogeneous pathophysiological state with high mortality, despite the development of cardiac intensive care units (CICUs) and the advanced treatments applied. The cornerstones of therapy that have been proposed in many algorithms are intravenous (i.v.) pressors and devices for mechanical circulatory support (MCS), depending on the CS profile (left, right, or biventricular involvement), etiology (acute myocardial infarction, heart failure, or other) and SCAI stage (A to E, with MCS generally recommended for Stages C–E). There are many gaps in the evidence regarding i.v. medications and devices, with the existing data being controversial. Moreover, there are differences in the devices’ availability and, as a result, a lack of experience in many centers. In this review article, an algorithm for the management of CS is proposed, and the gaps in every step are presented. Early clinical suspicion that leads to prompt diagnosis, health system organization, large-scale trials, and the configuration of national or regional shock centers could bridge the current therapeutic gaps and balance disparities in the management of CS in order to improve outcomes.

## 1. Introduction

Cardiogenic shock (CS) is a heterogeneous final pathophysiological state with potentially fatal consequences for patients admitted to cardiac intensive care units (CICUs) [1]. It has a rising prevalence and remains highly morbid, despite a steady, small decline in the adjusted trends of in-hospital mortality [1]. Acute coronary syndrome has been the most common etiology and the most studied type of CS in randomized control trials (RCTs), because 5–10% of cases of myocardial infarction (MI) result in CS [2]. The overall CS short-term mortality ranges from 30% to 40%, while the 1-year mortality may exceed 50% [3,4]. The economic impact of CS is important, especially in cases complicated with multiorgan failure, because of long CICU hospitalization and resource utilization, with the annual cost in the US exceeding $65 million [5,6]. Despite advances in treatment with early revascularization strategies and mechanical circulatory support (MCS) devices, RCTs have failed to present improved mortality [7]. Moreover, heart failure (HF) has emerged as the leading cause of CS in the past decade, with notable differences in patients’ baseline characteristics, comorbidities, and outcomes [8,9].

The early clinical recognition of CS is of utmost importance in order to initiate treatment immediately. Thorough physical examination and interpretation of biochemical markers (like serum lactate levels) play a crucial role in prognostication prior to invasive hemodynamic monitoring implementation. Patients with CS should be categorized according to (a) the underlying pathophysiology; (b) the severity, mainly according to the Society of Cardiovascular Angiography Interventions (SCAI) staging; and (c) the profile, i.e., the ventricle(s) affected (left, right, or biventricular shock), as these factors indicate the appropriate management and may alter outcomes [10]. The prognostication of CS patients is challenging, due to a lack of validated focused risk scores for this specific population [11]. Furthermore, therapeutic decisions may be affected by demographic, clinical, and logistic factors. Clinical questions arise regarding the proper use of MCS in CS concerning the time of initiation, escalation, and weaning or the type of device according to the profile, SCAI stage, and etiology [12].

The aim of the current review is to propose an algorithm for the management of patients with CS based on contemporary knowledge but also taking into account financial restrictions that prevent the widespread utilization of MCS, highlight gaps in the evidence, and emphasize the need for national-based treatment protocols and large-scale registries that will provide robust data to strengthen evidence-based clinical practice, guide resource distribution, and improve outcomes.

## 2. Definitions and Etiology of CS

### Definitions

**Figure 1:**
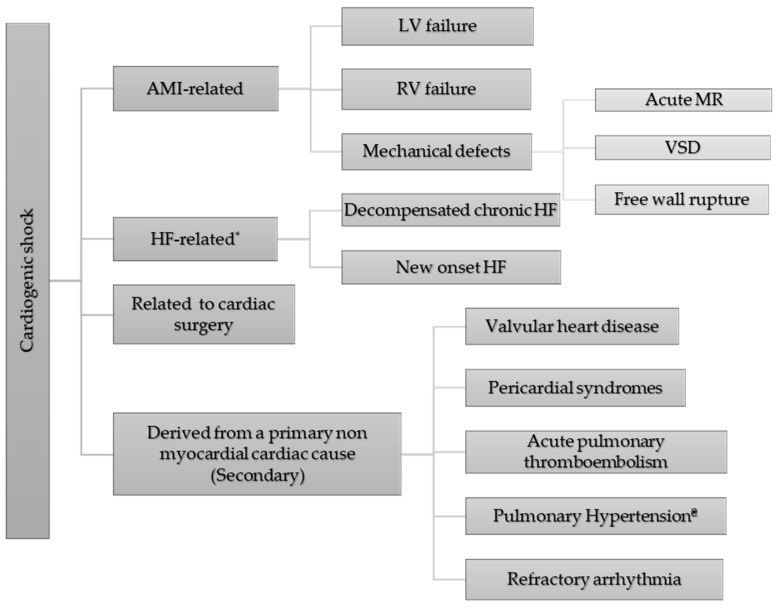
Schematic representation of the most frequent causes that lead to cardiogenic shock. In some cases, more than one causative factor may be present at the same time. Abbreviations: AMI = acute myocardial infarction, CMP = cardiomyopathy, HF = heart failure, LV = left ventricle, MR = mitral regurgitation, RV = right ventricle, VSD = ventricular septal defect. * = including acute myocarditis; stress CMP; peripartum CMP; alcoholic CMP; tachycardia-induced CMP, even if tachycardia may no longer exist; septic CMP; infiltrative/restrictive CMP; hypertrophic CMP; or dilated CMP, induced by drugs with negative inotropic effect, etc. ₴ = excluding group 2 pulmonary hypertension where pulmonary hypertension is caused by left ventricular dysfunction and would be classified as HF-related.

The definitions that are used most frequently with regard to the etiology of CS are (a) acute myocardial infarction-related CS (AMI-CS), which is used for the diagnosis of CS resulting from an AMI [either ST elevation myocardial infarction (STEMI) or non-ST elevation myocardial infarction (NSTEMI)] or its complications (acute bradyarrhythmia, high-degree heart block, tachyarrhythmia, or post-cardiac arrest); (b) HF-related CS (HF-CS), which is used for patients with a decompensation of a known chronic HF syndrome or an acute de novo presentation of HF with CS—patients with HF-CS are further subcategorized by the specific etiology (acute myocarditis, cardiomyopathies like takotsubo, peripartum, alcohol-related, tachycardiomyopathy, or infiltrative diseases); (c) post-pericardiotomy CS related to a cardiac surgery; and (d) secondary non-myocardial CS (severe valvular heart disease, pericardial disease, pulmonary embolism, or pulmonary hypertension) [13]. The main causes of CS are presented in Figure 1.

The currently prevailing categorization of CS is based on the SCAI stages [14]. Stage A: Patient is not currently experiencing signs or symptoms of CS but is at risk for its development. Stage B: Relative hypotension [systolic blood pressure (SBP) below 90 mmHg or mean arterial pressure (MAP) below 60 mmHg] or tachycardia but without hypoperfusion (normal blood lactate levels) and, if hemodynamic assessment is performed, cardiac index (CI) ≥ 2.2 L/min/m^2^ and pulmonary artery (PA) saturation ≥ 65%. Stage C: Hypoperfusion (lactate levels ≥ 2 mmol/L) requiring intervention (inotrope, vasopressor, or mechanical support) beyond volume resuscitation. These patients typically present with relative hypotension, CI < 2.2 L/min/m^2^, pulmonary capillary wedge pressure (PCWP) > 15 mmHg, cardiac power output (CPO) ≤ 0.6 W, or pulmonary artery pulsatility index (PAPI) > 1.85. Stage D: Similar to Stage C but failing to respond to initial interventions. Stage E: Cardiac arrest with ongoing cardiopulmonary resuscitation and/or needing support by multiple interventions [(and MCS/extracorporeal membrane oxygenation (ECMO)].

Finally, CS is divided according to profile into left ventricular (LV), right ventricular (RV), or biventricular (BiV) shock, depending on ventricle dysfunction [13].

## 3. A Proposed Algorithm for the Management of Cardiogenic Shock

### 3.1. SCAI Stage A

Stage A patients are usually patients with extensive anterior infarction or acute de novo HF but without hypotension or tachycardia. They are at risk for developing CS and should be thoroughly examined, even when normotensive [15,16]. Especially for HF patients, the development of CS may take place later during hospitalization, and physicians must stay vigilant. In such patients, in addition to physical exam at baseline and every 1–2 h (SBP and MAP measurement, heart rate estimation, cardiac and lung auscultation, and extremities palpation), a full evaluation should be performed with electrocardiogram (ECG) acquisition for ST deviation or arrhythmias; echocardiogram for left ventricle ejection fraction (LVEF) and filling pressures, RV systolic function, and valvular disease; laboratory markers at baseline, 2–8 h, and, if CS develops, every 8 h (complete blood count; renal, liver, and thyroid function; troponin; natriuretic peptides; and inflammation biomarkers); arterial blood gases (pH, PCO_2_, PaO_2_, and lactate bicarbonate); respiratory function; and mental status [10]. Repeated evaluation may reveal an insidious hypoperfusion state needing prompt action that possibly reduces mortality [10].

No intravenous (i.v.) pressors are needed at that stage. An intra-aortic balloon pump (IABP) may be used only in complex percutaneous coronary interventions (PCIs) in specific patients with reduced LVEF, as it is associated with a relative reduction in all-cause mortality compared to unsupported PCI [17].

### 3.2. SCAI Stage B

Stage B patients are at the beginning of CS, with clinical evidence of hemodynamic instability (hypotension, tachycardia), with normal lactate (<2 mmol/L) and mildly impaired renal or liver function, while signs of congestion may appear [10]. All the previously mentioned clinical, laboratory, imaging, and hemodynamic examinations must be conducted. Inopressors (preferably norepinephrine) [18] should be used for stabilization, nevertheless, in the lowest dose (0.05–1 μgr/kg/min) and for the shortest duration, due to the fact that they increase myocardial oxygen demand [6]. The intravenous medications used in CS and their doses and mechanism of action are presented in Table 1. The role of MCS is uncertain [19], but for patients with AMI-CS, IABP and Impella usage for complex PCIs (Rotablator use especially when LVEF is <20%) is proposed [20]. Furthermore, recently reported data favor the use of Impella in AMI-CS patients with low SBP, because it reduced mortality (for SBP lower than 82 mmHg) when compared with standard care alone (odds ratio [OR]: 0.34; 95% CI: 0.18–0.63; *p*  <  0.001) [21].

The MCS devices used in CS and their characteristics are presented in Table 2 for the LV and Table 3 for RV support.

### 3.3. SCAI Stage C

Stage C patients are in classic CS, with hypoperfusion, raised lactate (≥2 mmol/L), impaired renal and liver biochemistry, and hypotension requiring support with inotropes or MCS. All the previously mentioned clinical, laboratory, imaging, and hemodynamic examinations must be conducted. One inopressor can be used for hemodynamic stabilization (in the lowest dose and for the shortest duration) [6]. Pulmonary artery catheter (PAC; the most widely used catheter is Swan–Ganz), although not widely used, is strongly proposed for invasive hemodynamic monitoring, and values of CI < 2.2 L/min/m^2^ and PCWP > 15 mmHg are expected [22]. The recognition of the profile (LV, RV, or BiV involvement) is of utmost importance. For the LV phenotype, IABP, Impella CP, or Impella 5.5 can be used [23,24]. For RV or BiV phenotypes, Impella CP, Impella 5.5, or VP-ECMO should be used [24,25,26,27].

#### 3.3.1. SCAI Stage C Due to HF-CS

Initially, an inopressor can be used, and a PAC is inserted within 2 h after initial diagnosis. If hypoperfusion is not restored within 6 h (lactate levels < 2 mmol/L), IABP is inserted because it is the most widely accessible device (or Impella if available).

#### 3.3.2. SCAI Stage C Due to AMI-CS

Patients will be transferred directly to the cath-lab with immediate inopressor use. A PAC and an MCS device will be inserted; coronary angiography will follow, and, if indicated, ad hoc reperfusion will be performed. After the PCI and according to hemodynamic state, the MCS may be removed, and the patient will be transferred to the CICU (with the PAC).

### 3.4. SCAI Stage D

Stage D patients are deteriorating, with prominent hypoperfusion (lactate ≥ 4 mmol/L) and worsening renal and liver function. They are hypotensive and require support with increasing doses of two inotropes or MCS devices [10]. All the previously mentioned clinical, laboratory, imaging, and hemodynamic examinations must be conducted, and a PAC must be inserted (if not already in place). For the LV phenotype, Impella CP, Impella 5.5, or a left ventricular assist device (LVAD) for long-term support can be used [24,25,28]. For RV or BiV phenotypes, Impella 5.5 or VA-ECMO should be used [26,27].

### 3.5. SCAI Stage E

Stage E patients are at the extreme edge of the spectrum (‘extremis’) of CS, with continuous deterioration, severe hypoperfusion (lactate levels ≥ 8 mmol/L) and severe acidosis, multiorgan failure, and refractory hypotension or requiring support with three or more inotropes or MCS devices [10]. All the previously mentioned clinical, laboratory, imaging, and hemodynamic examinations must be conducted, and a PAC must be inserted (if not already in place). For all phenotypes, VA-ECMO (a circuit consisting of a centrifugal pump, an oxygenator, and cannulas for blood drainage and return) should be considered [10], as it supports both the heart and lungs, relieves the right ventricle, and improves hemodynamics while sustaining end-organ perfusion. In the presence of other non-reversible causes or comorbidities, weaning should be proposed at this stage.

**Figure 2:**
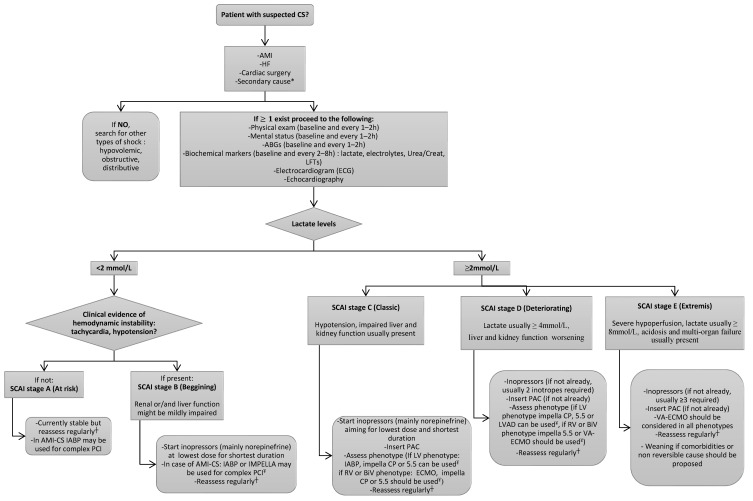
A proposed algorithm for the management of cardiogenic shock (CS). * As listed in Figure 1. ^†^ Regular reassessment should include at least physical examination (including mental status), ABGs, and biochemical markers. ^¥^ Device selection for MCS depends on available resources, local expertise, and hospital’s protocol (if existing). ABG = arterial blood gas, AMI = acute myocardial infarction, BiV = biventricular, CS = cardiogenic shock, HF = heart failure, IABP = intra-aortic balloon pump, LFTs = liver function tests, LV = left ventricle, MCS = mechanical circulatory support, PAC = pulmonary artery catheter, RV = right ventricle, VA-ECMO = veno-arterial extracorporeal membrane oxygenation, VP-ECMO = veno-pulmonary extracorporeal membrane oxygenation.

The proposed algorithm for CS treatment is presented in Figure 2.

In every SCAI stage, frequent revised assessment is needed to determine whether the patient is improving or deteriorating and whether further actions will be needed. If the patients are admitted to a hospital without a cardiac catheterization laboratory, CICU, percutaneous MCS (even IABP), or constant availability of such resources, they should be transferred to a referring institution with such capabilities, especially for AMI-CS and for patients at SCAI Stage C or higher.

## 4. Discussion

Despite the proposed algorithms for CS management, there are several gaps or even contradicting data concerning medications, interventional hemodynamic assessment, and MCS devices that could be used. First, the evidence regarding drug selection for circulatory support, complication rates, the standardized assessment of drug failure, and treatment targets in CS is limited and partly extrapolated from studies focusing on other shock entities [29]. The role of inotropes and vasoactive medication is ambiguous; they should be used for clinical stabilization, nevertheless, in the lowest dose and for the shortest duration, as they increase peripheral resistance and myocardial oxygen demand [30], and there is uncertainty with regard to their effect on mortality [31].

Vasoactive agents are proposed from SCAI Stage C in order to restore perfusion, although, in some cases, they can be initiated earlier in SCAI Stage B. Cardiologists are more familiar with noradrenaline, which is the most widely used inopressor, although retrospective analyses for dobutamine or milrinone show comparable outcomes [18,32]. On the contrary, another study failed to report any improvement in outcomes from the use of inotropes in CS patients; nevertheless, the evidence was of low quality [33]. Levosimendan was associated with a statistically significant reduction in short-term mortality compared to dobutamine (RR: 0.60, CI: 0.37–0.95), without a difference in the long-term mortality [34]. A clear indication, however, for noradrenaline is overlapping CS with systemic inflammation and vasoplegia [35]. Moreover, more than one vasopressor may be required, especially in the higher SCAI Stages D and E [30], because the combination of more than one agent in lower doses may reduce the possible interactions and adverse effects [13].

Furthermore, the implementation of the PAC in CS management is not well established and depends largely on the discretion of the attending physicians or the institutional resources [36]. In the present study, PAC use is strongly proposed for patients at Stage C or higher, because it confirms the LV and RV filling pressures; estimates the PAPI and CPO, thus determining the CS hemodynamic profile; and guides further therapeutic strategies [37]. A meta-analysis reported significantly lower in-hospital and 30-day mortality when a PAC was used (PAC: 4.6% to 41.5% vs control: 18.8% to 51.0%) (OR: 0.63, 95% CI: 0.41–0.97, I^2^ = 0.96), although no difference was found in the subgroup analysis (p-interaction = 0.83) [38]. Large RCTs are needed to strengthen the role of PACs in cardiac critical care, while other methods that have also been used for tissue perfusion monitoring, like cerebral oximetry (which detects cerebral hypoperfusion earlier than MAP changes) and renal tissue oximetry, which correlates strongly with mixed venous saturation and CO, are used even less [39,40].

The role of IABP in CS is still a matter of debate, because the long-term follow-up on all-cause mortality at 6 years showed no effect in the IABP-SHOCK II trial [41]. However, in many public national health system (NHS) hospitals worldwide, it is the only readily available MCS. In cases of AMI-CS, the use of the IABP was found to improve survival compared to reperfusion and pressors only [42], while in cases of HF-CS, patients with IABPs were more likely to have a favorable outcome than in those with AMI-CS [43], probably because of the proportionally greater cardiac output improvement. Nevertheless, the guidelines recommend against the routine use of IABP for AMI-CS, and its wide ongoing use is likely driven by its relatively low cost, its familiarity, the unavailability of other MCSs, the occurrence of mechanical complications from an AMI, or its use in patients with HF-CS [44]. Especially for HF-CS patients, there is a lack of consensus between the European (ESC) and American (ACCF/AHA) guidelines on the use of IABP, with the former stating that it may be considered (class IIb) and the latter characterizing it as useful (class IIa) [45]. In the present algorithm, IABP use has a place in all stages only when there is a shortage of other devices, as it is clearly recognized that the MCS device should be chosen based on the CS etiology and profile; at SCAI Stages A and B, IABP is proposed solely in AMI-CS and complex PCIs (at SCAI Stage B, Impella is preferred). At Stage C, IABP is proposed in both AMI-CS and HF-CS in the LV profile if Impella is unavailable, while for RV and BiV profiles, at SCAI Stage C or at higher stages (D–E), it is proposed only when the institutional resources lack other more indicated MCS devices.

The use of Impella showed a significant reduction in 180-day mortality among patients with STEMI and AMI-CS compared to standard of care (45.8% vs. 58.5%, hazard ratio: 0.74; 95% confidence interval: 0.55 to 0.99; *p* = 0.04) but higher complication rates (mainly bleeding and limb ischemia) [46]. A meta-analysis, however, reported that Impella use was related to significantly higher mortality (57% vs. 46%; OR: 1.44, 95% CI: 1.29–1.60; *p* < 0.001) and major bleeding (30% vs. 15%; OR: 2.93, 95% CI: 1.67–5.13; *p* < 0.001) compared to IABP in AMI-CS patients [47]. Whether the benefits outweigh the risks for centers less experienced with microaxial flow pumps is still uncertain [48]. Better patient selection and avoiding Impella implantation in futile situations or in possible lower-risk CS might be necessary to elucidate the possible advantages of Impella in future studies [49]. In the present algorithm, the use of Impella is clearly proposed (if available) over IABP for Stage B patients with AMI-CS that need a complex PCI and at higher stages (C–E) when other devices like VA-ECMO or LVAD are unavailable, irrespective of the etiology of CS (AMI or HF) or the profile (LV, RV, or BiV).

The utilization of VA-ECMO has been proposed for the higher SCAI stages of CS (stages D–E) irrespective of the phenotype, even as a bridge to heart transplantation [10,50]. Nevertheless, RCTs have not always shown better outcomes from the use of VA-ECMO compared to standard of care [45]. There are reports showing that VA-ECMO does not improve 30-day all-cause mortality in patients with AMI-CS; nevertheless, there may be a significant reduction in all-cause mortality at 12 months [27]. The largest trial, Extracorporeal Life Support in Cardiogenic Shock (ECLS-SHOCK), with 417 SCAI Stage C to E CS patients, reported the same all-cause mortality at 30 days and more complications (vascular, bleeding, stroke, or systemic embolism) in the VA-ECMO arm. The neutral result might be explained by several factors (device complications, increased LV afterload, higher risk of post-cardiac arrest hypoxic ischemic brain injury, and low rates of transition to destination therapies compared to other temporary MCS trials) [51]. Another matter of debate is the timing of initiation with regard to effectiveness. On the one hand, immediate implementation of VA-ECMO in patients with rapidly deteriorating or severe CS did not improve clinical outcomes compared to an early conservative strategy and staged ECMO use [52], but, on the other hand, a large study with 8619 patients reported lower in-hospital mortality when VA-ECMO was inserted within the first 24 h in CS patients at lower SCAI stages, without prior cardiac arrest, and in patients without previous MCS devices [53]. Although ECMO implementation remains controversial and a personalized approach is required with thorough evaluation of patient-related factors, in this proposed algorithm, the use of ECMO is clearly proposed (if available) over Impella for RV or BiV profiles at Stage C and also at Stages D–E for all profiles. Moreover, some institutions still use a setup of a left atrial drain to femoral circulation through an ECMO circuit or CentriMag like a TandemHeart, but the formal machine is no longer used, and it is not proposed in this algorithm [54].

As mentioned already, the outcome of patients admitted with CS is affected by several components, e.g., patient-related factors and characteristics (etiology, low LVEF, lactate levels, previous reperfusion treatment, and comorbidities) [55] and factors related to the organization of the health system, cooperation between regional health institutions and fully equipped CS centers, geography (remote regions, island regions), financial constraints, and resource allocation [56,57]. Patients treated in the US had significantly lower 30-day and 1-year mortality compared to those treated in other countries in an older report, probably due to the greater use of invasive diagnostic and therapeutic interventions [58].

Given the fact that health resources are limited, priority setting and resource allocation within the context of publicly funded health care systems are essential [59]. The organization of NHSs should be pyramidal: (a) a few first-level, fully equipped shock centers with CICUs, MCSs, long-term LVADs, and even heart transplantation capabilities; (b) enough second-level, peripheral institutions with CICUs, cath-labs, MCSs, and hemodynamic monitoring; and (c) widespread third-level centers capable of CS diagnosis that may only be staffed with general cardiologists, without cath-labs or CICUs, that will need to refer patients to the next level [10]. National-based treatment protocols and large-scale registries that will provide robust data on CS occurrence are needed to define needs more accurately and formulate policies. Toward that direction, artificial intelligence (AI) could also be used through supervised machine learning (ML) techniques for early identification, monitoring, and outcome prediction and through unsupervised ML-assisted phenotyping of CS patients [12,60].

The development of CICUs has improved outcomes [61], and staffing them with cardiac intensivists has led to a further reduction in mortality rates [62]. A retrospective study of patients presenting with CS reported that in-hospital mortality was lower in CICUs with a cardiac intensivist than those without an intensivist (25.4 vs. 40.1%), while if ECMO was used, the mortality rates were lower (38.0% vs. 62.2%), the dopamine use was lower, and the norepinephrine use was higher in the cardiac intensivist group [63]. Similarly to general ICUs, where patients who are admitted fulfill specific criteria [64], patients with CS admitted to CICUs should also be carefully selected for treatment escalation so that resources will not be spent in futile cases [49]. Professionals’ ability to distinguish between resource limitations and futility should be enhanced by medical policies to promote legitimacy in end-of-life decision-making [65].

Another matter of discussion is sepsis, which can lead to sepsis-induced cardiogenic shock (SICS) due to secondary myocardial dysfunction (reversible or not), even when loading conditions are restored [66]. SICS has an even greater mortality [67], and invasive hemodynamic monitoring with a PAC, for optimal volume restoration, is necessary. MCS might also be considered because high-dose vasopressors and inotropes (associated with worse outcomes) may be required [66,68]. Whether CICUs or general ICUs are preferable for the optimal care of SICS is based largely on institutional organization.

Finally, large-scale clinical trials designed for research on CS will assist in more appropriate population selection and improve statistical analysis [69]. The configuration of national or regional shock teams and centers and prognostication tools could improve the management of patients with CS.

## 5. Summary and Future Recommendations

CS is a diverse pathophysiological state with high mortality. The outcome of patients is affected by several factors, both patient-related (cause, phenotype, and comorbidities) and system-related (mainly organization and resource availability). There are many gaps in the evidence regarding intravenous medication, without a clear supremacy of one pressor over the others. The same applies for MCS devices, probably because of the lack of robust criteria for their initiation and the lack of experience, which might increase complications. Unavoidably, a proposed algorithm for CS management should take these limitations into account. Furthermore, clinical suspicion that leads to prompt diagnosis and treatment may improve outcomes. Large-scale RCTs and configuration of national or regional shock centers could shed light on current gaps by depicting the realistic prevalence and the unmet needs of these critically ill patients.

## Figures and Tables

**Table 1:** The intravenous medications used in CS.

Class	Agent	Receptors	Dosage	Hemodynamic Effect	Half Life	Adverse Effects
Inotropes	Dobutamine	β1 > β2 > α	2–20 μg/kg/min	↑CO, ↓SVR, ↓PVR ↓LVFP	2–3 min	Atrial and ventricular arrhythmias, Tachyphylaxis
	Dopamine	low doses: D1 intermediate: β1 high: a	1–3 μg/kg/min 3–10 μg/kg/min >10 μg/kg/min	↑CO ↑↑CO, ↑SVR ↑↑SVR, ↑CO	2 min	Tachycardia, AF, PVCs Myocardial ischemia
Vasopressors	Noradrenaline	α >> β1 > β2	0.05–0.4 μg/kg/min	↑↑SVR, ↑CO	3 min	Peripheral ischemia (renal, splanchnic)
	Adrenaline	β1 = β2 > α	0.01–0.5 μg/kg/min	↑↑CO, ↑↑SVR	2 min	Ventricular arrhythmias, decreased splanchnic blood flow
	Vasopressin	V1 and V2 in smooth muscle	0.02–0.04 U/min	↑↑SVR	10–20 min	Abdominal pain, confusion, angina
	Phenylephrine	a1	0.1–10 μg/kg/min	↑↑SVR, ↓CO	5 min	Myocardial ischemia
Calcium sensitizer	Levosimendan		0.05–0.2 μg/kg/min	↑CO, ↓SVR, ↓PVR	1 h–8 h	Hypotension, arrhythmias
PDE3 inhibitor	Milrinone	PDE3	0.125–0.75 μg/kg/min	↑CO, ↓SVR, ↓PVR	2 h	Hypotension, atrial and ventricular arrhythmias
Chronotrope/ inodilator	Isoproterenol	β1 and β2	2–20 μg/min	↑↑CO, ↓SVR, ↓PVR	2.5–5 min	Sinus tachycardia and other arrhythmias, hypotension, angina, flushing, headaches

Abbreviations. AF: atrial fibrillation, CO: cardiac output, LVFP: left ventricular filling pressure, PDE3: phosphodiesterase, PVCs: premature ventricular contractions, PVR: pulmonary vascular resistance, SVR: systemic vascular resistance. ↑increase, ↑↑bigger increase, ↓decrease.

**Table 2:** Mechanical circulatory support systems for the left ventricle.

	IABP	Impella CP or 5.5	TandemHeart	VA-ECMO
Cardiac flow	0.5–1 L/min	2–5.5 L/min	2.5–5 L/min	5–7 L/min
Blood flow	Not applicable	LV to Ao	LA to femoral artery	RA to femoral artery
LV afterload	Decrease	Neutral	Increase	Marked increase
MAP	Increase	Marked increase	Marked increase	Marked increase
Cardiac power	Increase	Marked increase	Marked increase	Marked increase
Venous access	None	None	Yes	Yes
Indications	ADHF, high-risk PCI, AMI with <TIMI 3 flow post-PCI, post-cardiotomy CS	ADHF, CS, refractory malignant arrhythmias, high-risk PCI	ADHF, CS, refractory malignant arrhythmias, high-risk PCI	ADHF, CS, massive PE, cardiac arrest, refractory malignant arrhythmias
Contraindications	Severe PAD, AAA, significant AR	LV thrombus, mechanical AV, severe PAD	VSD, significant AR, LA thrombus	Severe PAD, significant AR, aortic dissection
Support provided	Minimal hemodynamic support	Partial LV support: 2.5 and CP Complete LV support: 5.0, 5.5	Partial to complete LV support based on the size of the outflow (arterial) cannula	Complete BiV support
Complications	Limb ischemia, vascular injury, thromboembolism, bleeding, stroke, balloon leak/rupture	Bleeding, limb ischemia, thromboembolism, vascular injury, stroke	Bleeding, limb ischemia, thromboembolism, vascular injury, stroke, residual atrial septal defect	Bleeding, limb ischemia, thromboembolism, vascular injury, stroke
Recommended max use	14 days	10 days	14 days	14 days

Abbreviations: AAA: ascending aortic aneurysm, ADHF: acute decompensated heart failure, AMI: acute myocardial infarction, Ao: aorta, AR: aortic regurgitation, AV: aortic valve, BiV: biventricular, LA: left atrium, LV: left ventricle, PA: pulmonary artery, PAD: peripheral artery disease, PCI: percutaneous coronary intervention, PE: pulmonary embolism, RA: right atrium, RV: right ventricle, TR: tricuspid regurgitation, VSD: ventricular septal defect.

**Table 3:** Mechanical circulatory support systems for the right ventricle.

	Impella RP	TandemHeart (RA-PA)	VA-ECMO
Cardiac flow	Max 4 L	Max 4 L	3–7 L/min
Blood flow	Femoral vein to PA	From RA to PA	RA to femoral artery
LV afterload	No change/Increase	No change	Marked increase
MAP	Increase	Increase	Marked increase
Cardiac power	No change/Increase	No change/Increase	Marked increase
Venous access	Yes	Yes	Yes
Indications	Acute RV failure, RV infarct with shock, post-cardiotomy RV failure, post-cardiac transplantation	RV failure when Impella not available	ADHF, CS, massive PE, cardiac arrest, refractory malignant arrhythmias
Contraindications	Severe TR, severe PH, severe LV dysfunction	Severe PH, severe LV dysfunction	Severe PAD, significant AR, aortic dissection
Support provided	RV support	RV support	Complete BiV support
Complications	Arrhythmias, vascular injury, bleeding, thrombosis, embolic events, device migration/malfunction	Major bleeding at large venous sites, thromboembolism, hemolysis, circuit-related infection, RV suction events	Bleeding, limb ischemia, thromboembolism, vascular injury, stroke
Recommended max use	14 days	14 days	14 days

Abbreviations: AAA: ascending aortic aneurysm, ADHF: acute decompensated heart failure, AMI: acute myocardial infarction, Ao: aorta, AR: aortic regurgitation, AV: aortic valve, BiV: biventricular, LA: left atrium, LV: left ventricle, MAP: mean arterial pressure, PA: pulmonary artery, PAD: peripheral artery disease, PCI: percutaneous coronary intervention, PE: pulmonary embolism, RA: right atrium, RV: right ventricle, TR: tricuspid regurgitation, VSD: ventricular septal defect.

## Data Availability

Not applicable.

## Abbreviations

The following abbreviations are used in this manuscript:

AI	artificial intelligence
AMI	acute myocardial infarction
AMI-CS	acute myocardial infarction-related cardiogenic shock
BiV	biventricular
CI	cardiac index
CICU	cardiac intensive care unit
CPO	cardiac power output
CS	cardiogenic shock
ECG	electrocardiogram
ECMO	extracorporeal membrane oxygenation
HF	heart failure
HF-CS	heart failure-related cardiogenic shock
IABP	intra-aortic balloon pump
LV	left ventricle
LVAD	left ventricular assist device
LVEF	left ventricular ejection fraction
MAP	mean arterial pressure
MCS	mechanical circulatory support
MI	myocardial infarction
NHS	national health system
NSTEMI	non-ST elevation myocardial infarction
PA	pulmonary artery
PAC	pulmonary artery catheter
PAPI	pulmonary artery pulsatility index
PCI	percutaneous coronary intervention
PCWP	pulmonary capillary wedge pressure
RCT	randomized controlled trial
RV	right ventricle
SBP	systolic blood pressure
SCAI	Society for Cardiovascular Angiography and Interventions
SICS	sepsis-induced cardiogenic shock
STEMI	ST elevation myocardial infarction
VA-ECMO	veno-arterial extracorporeal membrane oxygenation
